# Predictive factors for early neurological deterioration after intravenous thrombolysis of single subcortical infarction in the territory of the middle cerebral artery

**DOI:** 10.1002/brb3.3283

**Published:** 2023-10-17

**Authors:** Meng Zhao, Xuemin Zhong, Jiaxiu Du, Lanying He, Jian Wang

**Affiliations:** ^1^ Department of Neurology Zhengzhou People's Hospital Zhengzhou China; ^2^ Department of Neurology The Second People's Hospital of Chengdu Chengdu China

**Keywords:** early neurological deterioration, middle cerebral artery, perforating artery, single subcortical infarction

## Abstract

**Introduction:**

Patients with a single subcortical infarction (SSI) in the territory of the middle cerebral artery (MCA) often experience early neurological deterioration (END) despite receiving intravenous thrombolytic therapy (IVT). In this study, predictors of END were investigated in patients with SSI in the MCA after IVT.

**Methods:**

Patients with SSI in the MCA territory who had received IVT between June 2020 and 2022 were included. END was defined as an increase in the total National Institutes of Health Stroke Scale (NIHSS) score by ≥2 or in the motor NIHSS score by ≥1 within the first 72 h of admission. Patients with proximal (pSSI) and distal SSI (dSSI) were analyzed to determine SSI type‐specific predictors for END.

**Results:**

We evaluated 174 patients with SSI in the MCA territory who underwent IVT. Multivariable logistic regression analysis showed that pSSI (odds ratio [OR] = 0.242; 95% confidence interval [CI], 0.104–0.564; *p* = .001), lower high‐density lipoprotein cholesterol (HDL‐C) (OR = 0.150; 95% CI, 0.033–0.682; *p* = .014), higher blood glucose (OR = 0.858; 95% CI, 0.752–0.979; *p* = .023), and higher red blood cells count (OR = 1.966; 95% CI, 1.154–3.349; *p* = .013) were risk factors for END. In patients with pSSI, HDL‐C and blood glucose were associated with END. No variable related to END was found in the dSSI group.

**Conclusions:**

The proportion of END in patients with SSI in the MCA territory after IVT was not low; therefore, pSSI, HDL‐C, blood glucose, and red blood cells should be monitored closely. The frequency and predictors of SSI in the MCA territory differed between pSSI and dSSI.

## Graphical abstract

Patients with a single subcortical infarction (SSI) in the territory of the middle cerebral artery (MCA) often experience early neurological deterioration (END) despite receiving intravenous thrombolytic therapy (IVT). In this study, predictors of END were studied in patients with SSI in the MCA after IVT.

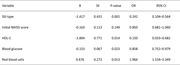

## 1 INTRODUCTION

Single subcortical infarctions (SSIs) are infarctions in the territory of perforating arteries with a relatively favorable prognosis (Del Bene et al., 2012; Fisher, 1965; Jiang et al., 2020; Ois et al., 2008; Yamada et al., 2004). However, early neurological deterioration (END), observed in the acute phase of infarction that leads to unexpected and severe disability, is common in these patients (Hallevi et al., 2012; He et al., 2019; Park et al., 2016; Yamamoto et al., 2010). Intravenous thrombolytic therapy (IVT) remains the primary choice of treatment for SSI within the thrombolysis time window, although some controversies exist regarding the use of thrombolysis in the small pawn (National Institutes of Health Stroke Scale [NIHSS] score ≤3) (IST‐3 Collaborative Group, 2012; National Institute of Neurological Disorders and Stroke rt‐PA Stroke Study Group, 1995; Wu et al., 2020). However, studies have shown that despite receiving IVT, a subset of these patients still present with END, which can affect their long‐term functional outcomes (He et al., 2019; Park et al., 2016; Zhou et al., 2018). Therefore, identifying presentation features that predict END is important to help screen high‐risk patients. In clinical practice, SSI in the middle cerebral artery (MCA) territory is more common than in the posterior circulation supply area (Del Bene et al., 2012; Tamura et al., 2013; Yamada et al., 2004). This study aimed to identify the predictive factors for END in patients with SSI in the MCA territory after IVT.

## 2 MATERIALS AND METHODS

### 2.1 Ethics

This study received ethical approval from the Ethics Committee Review Boards of Chengdu Second People's Hospital, which waived the requirement for written informed consent because of its retrospective design and use of de‐identified and anonymized patient information. All experiments were performed according to the relevant guidelines and regulations.

### 2.2 Study population

We included patients with SSI in the MCA territory who had received IVT between June 2020 and 2022 at Zhengzhou People's Hospital and Chengdu's Second People's Hospital. All patients were carefully evaluated for stroke using brain magnetic resonance imaging (MRI), magnetic resonance angiography (MRA), computed tomography angiography (CTA), echocardiography, electrocardiography (ECG), and laboratory examinations. The inclusion criteria for the study were as follows: age >18 years, the duration between symptom onset and admission time <6 h (time of symptom onset was taken as the time when the patient last appeared normal), diffusion‐weighted imaging (DWI) conducted on admission before IVT, acute isolated infarction located in the MCA perforating territory, the largest diameter of the lesion on axial DWI <20 mm, and IVT. The exclusion criteria were as follows: patients treated with endovascular interventional therapy, significant stenosis (>50%) in the extracranial internal carotid artery, and potential cardioembolic sources (atrial septal defect found on echocardiography or atrial fibrillation on ECG).

### 2.3 Clinical assessment

The detailed demographic and clinical parameters of the patients were recorded upon admission. The parameters included age, sex, history of hypertension (receiving antihypertensive treatment or blood pressure ≥140/90 mmHg upon repeated measurements), diabetes mellitus (receiving medications for diabetes mellitus, fasting blood glucose ≥7.0 mmol/L, 2‐h postprandial blood glucose or random blood glucose ≥11.1 mmol/L), hyperlipidemia (receiving cholesterol‐reducing agents or overnight fasting cholesterol level ≥5.17 mmol/L), smoking (current smoker or quit smoking ≤6 months prior), alcohol consumption (>80 g/day or quit drinking ≤6 months prior), and systolic and diastolic blood pressure on admission and after IVT. The initial severity of stroke was rated using the NIHSS score and subsequently measured daily from admission to discharge by two neurologists who were not otherwise involved in this study. Laboratory examinations, including blood glucose profiles, lipid profiles, white blood cell counts and distributions, and clotting‐related indicators, were performed within the first 24 h of admission. As a major outcome, END was defined as an increase in the total NIHSS score by ≥2 or in the motor NIHSS score by ≥1 within the first 72 h of admission.

### 2.4 Radiological assessment

In this study, all participants underwent DWI of the brain on admission and before administration of the IVT, a brain MRI on admission (T1WI, T2WI, and fluid‐attenuated inversion recovery were included in the imaging protocol), and CTA or MRA (3.0‐T, Signa EchoSpeed; GE Healthcare) within 24 h of admission. Based on previous research, SSI was defined as a single lesion invading a perforating artery of the MCA, basilar artery, or posterior cerebral artery territories, such as the striatocapsular, thalamic, or brain stem areas (Del Bene et al., 2012; Nam et al., 2021; Yamada et al., 2004). Only a single lesion that invaded the perforating artery of the MCA was studied. According to a previous study, DWI‐defined SSI lesion patterns were divided into proximal SSI (pSSI) and distal SSI (dSSI) based on the relationship between the location of the lesion and the parent artery (Duan, Fu, et al., 2015; Nam et al., 2021; Yamamoto et al., 2010). A pSSI pattern was defined as an infarction located in the proximal region near the parent artery (extending to the basal surface of the parent artery), whereas a dSSI pattern was defined as an infarction located in a more distal area (not extending to the basal surface of the parent artery) (Del Bene et al., 2012; Duan, Fu, et al., 2015; Yamada et al., 2004; Zhou et al., 2018). The largest infarction on axial DWI was also analyzed. Two investigators defined the categorization of SSI. In the case of a dispute, a unanimous decision was reached through consultation.

### 2.5 Statistical analyses

All statistical analyses were performed using SPSS (version 27.0; IBM Corp.). The Mann–Whitney *U* test was conducted for the univariate analyses to identify possible predictors of END for continuous variables and Chi‐squared or Fisher's exact tests for categorical variables (Nam et al., 2021). According to the results of univariate analyses, variables with an associated *p*‐value of <.05 were introduced into the multivariable logistic regression analysis. To study the differences in predictive factors for END, according to the location of the SSI, we performed a subgroup analysis by SSI type (Nam et al., 2021). Univariate analysis through simple logistic regression analysis in each subgroup (pSSI vs. dSSI) was performed, and variables with *p* < .05 were introduced into the multivariable logistic regression analysis (Duan, Fu, et al., 2015; Nam et al., 2021).

## 3 RESULTS

A total of 175 patients with SSIs in the territory of the MCA receiving IVT were evaluated (mean age = 61.8 years; 65.1% male; mean initial NIHSS score = 3; mean door–needle time [DNT] = 21 min; NIHSS score after IVT = 2; modified Rankin Scale score after IVT = 1). END occurred in 51 (29.1%) patients, and the median time of admission was 10.5 (1−71.5) h. Furthermore, 103 (58.7%) and 71 (41.8%) patients had SSI lesions in the distal and proximal regions, respectively. Tables 1 and 2 present descriptions of the baseline characteristics and comparisons according to the SSI type.

**TABLE 1:** Baseline characteristics of patients with and without END.

Variable	Non‐END (*n* = 124)	END (*n* = 51)	*p*‐value
Age, years (IQR)	64.5 (55−71.75)	59 (54−70)	.187
Sex, male (%)	77 (62.1)	37 (72.5)	.207
Hypertension history, *n* (%)	63 (50.8)	32 (62.7)	.150
Diabetes history, *n* (%)	41 (33.1)	18 (35.3)	.777
Hyperlipidemia, *n* (%)	57 (46)	25 (49)	.410
Smoking history, *n* (%)	31 (25)	15 (29.4)	.547
Alcohol consumption history, *n* (%)	29 (23.8)	14 (27.5)	.570
DNT (min)	21 (15−28.8)	21 (14−27)	.455
Initial NIHSS score	3 (1−4)	4 (2−5)	.012
SBP on admission (mmHg) (SD)	154.5 (138−168)	155 (140−170)	.522
DBP on admission (mmHg)	85 (77−95)	87 (80−97)	.275
Infarction size on axial DWI (mm^2^)	137.63 (64.97−241.57)	184.7 (108.1−400.3)	.014
Total cholesterol (TC), mmol/L	4.52 (3.52−5.1)	4.30 (3.39−5.18)	.574
Triglyceride (TG), mmol/L	1.43 (0.88−1.97)	1.42 (0.93−2.45)	.988
HDL cholesterol, mmol/L	1.16 (0.97−1.35)	1.03 (0.89−1.21)	.004
LDL cholesterol, mmol/L	2.37 (1.69−3.02)	2.53 (1.73−3.14)	.516
Blood glucose, mmol/L	6.87 (5.84−10.09)	6.17 (5.54−8.74)	.044
Red blood cells, ×10^12^/L	4.57 (4.16−4.84)	4.70 (4.34−5.33)	.007
White blood cells, ×10^9^/L	7.53 (5.75−9.7)	7.98 (5.77−9.55)	.618
Neutrophil counts, ×10^9^/L	5.19 (3.66−7)	4.91 (3.79−6.95)	.749
Neutrophil‐to‐lymphocyte ratio	2.98 (1.81–6.24)	3.13 (1.84–4.34)	.592
SBP difference before and after thrombolysis, mmHg	12 (−5.75 to 29)	14 (5−24)	.450
DBP difference before and after thrombolysis, mmHg	7 (−4 to 18.75)	8 (0−16)	.723
SSI type, *n* (%)			<.001
Proximal SSI	40 (32.3)	32 (62.7)	
Distal SSI	84 (67.7)	19 (37.3)	

Abbreviations: END, early neurological deterioration; DBP, diastolic blood pressure; DNT, door–needle time; DWI, diffusion‐weighted imaging; HDL, high‐density lipoprotein; IQR, interquartile range; LDL, low‐density lipoprotein; NIHSS, National Institutes of Health Stroke Scale; SBP, systolic blood pressure; SSI, single subcortical infarction.

**TABLE 2:** Multivariable logistic regression analysis of possible predictors of END.

Variable	*B*	*SE*	*p*‐value	OR	95% CI
SSI type	−1.417	0.431	.001	0.242	0.104−0.564
Initial NIHSS score	−0.163	0.113	.149	0.850	0.681−1.060
HDL‐C	−1.894	0.771	.014	0.150	0.033−0.682
Blood glucose	−0.153	0.067	.023	0.858	0.752−0.979
Red blood cells	0.676	0.272	.013	1.966	1.154−3.349

Abbreviations: CI, confidence interval; END, early neurological deterioration; HDL‐C, high‐density lipoprotein cholesterol; OR, odds ratio; NIHSS, National Institutes of Health Stroke Scale; SSI, single subcortical infarction.

In the univariate analysis, END was significantly associated with a higher initial NIHSS score, blood glucose level, and red blood cell count; a lower HDL‐C level; and the presence of pSSI lesion (Table 1).

According to the multivariable logistic regression analysis, the pSSI type (odds ratio [OR] = 0.242; 95% confidence interval [CI], 0.104−0.564; *p* = .001), lower HDL‐C (OR = 0.150; 95% CI, 0.033−0.682; *p* = .014), higher blood glucose (OR = 0.858; 95% CI, 0.752−0.979; *p* = .023), and higher red blood cells count (OR = 1.966; 95% CI, 1.154−3.349; *p* = .013) were associated with END.

In the subgroup analysis performed according to the SSI type, a difference between the predictors of END was observed between patients with dSSI and those with pSSI (Table 3). In patients with pSSI, the initial NIHSS score, blood glucose level, HDL‐C level, and prothrombin time were significantly associated with END in the univariate analysis. However, in patients with dSSI, infarction size on axial DWI (mm^2^), triglyceride, and HDL‐C were significantly associated with END in the univariate analysis. In multivariable logistic regression analysis, pSSI, lower HDL‐C, and higher blood glucose levels were significantly associated with END; however, in patients with dSSI, no predictive factor was associated with END (Table 4).

**TABLE 3:** Baseline characteristics of patients according to SSI type.

	Proximal SSI (*n* = 72)			Distal SSI (*n* = 103)		
Variable	Non‐END (*n* = 40)	END (*n* = 32)	*p*‐value	Non‐END (*n* = 84)	END (*n* = 19)	*p‐*value
Age, years	65 (65.58 ± 10.90)	56 (58.66 ± 10.10)	.21	64(62.50 ± 12.66)	65(64.84 ± 10.59)	.499
Sex, male	22 (50)	22 (50)	.234	55 (78.57)	15 (21.43)	.196
Hypertension history, *n* (%)	17 (46)	20 (54)	.092	40 (83.33)	8 (16.67)	.663
Diabetes history, *n* (%)	12 (57.1)	9 (42.9)	.862	29 (76.32)	9 (23.68)	.295
Hyperlipidemia, *n* (%)	18	23	.775	40	17	.586
Smoking history, *n* (%)	1 (100)	0 (0)	.368	23 (82.14)	5 (17.86)	.925
Alcohol consumption history, *n* (%)	8 (44.4)	10 (55.6)	.273	20 (80)	5 (20)	.818
DNT (min)	19.5 (13.3−29.5)	17 (14.3−27.3)	.586	21.5 (15−28.8)	22 (13−27)	.939
Initial NIHSS score	3 (2−6)	4 (2.3−7.5)	.039	2 (1−4)	3 (2−4)	.143
SBP on admission (mmHg) (SD)	157 (141.25−174.75)	155 (139.25−166.25)	.468	154 (136−164.75)	156 (143−172)	.219
DBP on admission (mmHg) (SD)	87.5 (79−98.5)	89.5 (80.5−97.00)	.533	84 (75.25−94)	85 (79−95)	.730
infarction size on axial DWI (mm^2^)	203.57 (62.94−472.42)	169.18 (96.31−386.37)	.928	116.95 (65.5−194.05)	240.02 (108.09−409.4)	.009
Total cholesterol, mmol/L, (SD)	4.54 (3.34−5.74)	4.09 (3.27−5.12)	.170	4.45 (3.56−5.03)	4.71 (3.71−5.32)	.363
Triglyceride, mmol/L, (SD)	1.34 (0.79−1.90)	1 (0.79−1.51)	.321	1.44 (0.97−1.99)	2.04 (1.42−2.64)	.030
HDL cholesterol, mmol/L, (SD)	1.18 (1.02−1.46)	1.04 (0.92–1.22)	.024	1.14 (0.96−1.29)	0.96 (0.8−1.15)	.020
LDL cholesterol, mmol/L, (SD)	2.38 (1.48−3.17)	2.39 (1.62−3.13)	.932	2.35 (1.81−2.97)	2.73 (2.12−3.51)	.179
Blood glucose, mmol/L (SD)	7.83 (6−11.43)	5.84 (5.47−7.16)	.003	6.6 (5.7−8.84)	7.26 (5.58−9.81)	.779
Prothrombin time	11.50 (11.03−12.28)	12.05 (11.7−12.5)	.034	11.70 (11.2−12.50)	12.10 (10.9−12.20)	.779
Activated partial thromboplastin time	27.05 (54.65−28.80)	25.65 (23.93−27.25)	.097	25.85 (24.4−28.48)	26.80 (24.4−29.10)	.677
Red blood cells, ×10^12^/L	4.64 (4.08−5.01)	4.69 (4.37−5.30)	.072	4.54 (4.21−4.75)	4.70 (4.21−5.77)	.111
White blood cells, ×10^9^/L	7.76 (6.22−8.85)	7.21 (5.29−8.92)	.279	7.36 (5.59−9.93)	8.41 (5.93−10.98)	.578
Neutrophil counts, ×10^9^/L	5.17 (3.67−6.85)	4.79 (3.51−6.28)	.541	5.21(3.66−7.64)	5.72 (4.50−8.05)	.557
SBP difference before and after IVT, mmHg (SD)	16 (−2.75 to 32.75)	16 (4.25−25.50)	.905	11.00 (−7.5 to 28.25)	12 (5−20)	.753
DBP difference before and after IVT, mmHg (SD)	7.50 (−3 to 15.75)	9.50 (0.5−16.75)	.518	6.50 (−5.75 to 21.75)	6 (0−16)	.956

Abbreviations: DBP, diastolic blood pressure; DNT, door‐needle time; END, early neurological deterioration; HDL, high‐density lipoprotein; IVT, intravenous thrombolytic therapy; LDL, low‐density lipoprotein; NIHSS, National Institutes of Health Stroke Scale; SBP, systolic blood pressure; SSI, single subcortical infarction.

**TABLE 4:** Multivariable logistic regression analysis of possible predictors of early neurological deterioration according to the SSI type.

Variable	Proximal SSI			Distal SSI		
	*p*‐value	OR	95% CI	*p*‐value	OR	95% CI
Initial NIHSS	.057	0.722	0.516−1.010	–	–	–
HDL‐C	.031	0.092	0.011−0.800	.114	0.173	0.020−1.521
Blood glucose	.019	0.791	0.650−0.963	–	–	–
PT	.071	1.896	0.946−3.801	–	–	–
TG	–	–	–	.504	1.103	0.827−1.470

Abbreviations: HDL‐C, high‐density lipoprotein cholesterol; NIHSS, National Institutes of Health Stroke Scale; PT, prothrombin time; SSI, single subcortical infarction; TG, triglyceride.

## 4 DISCUSSION

In this study, END occurred in 29.1% of patients with SSI in the MCA territory despite receiving IVT. To our knowledge, this is the only study focusing on SSI after IVT. Previous studies on SSI without IVT have reported rates between 11.9% and 43%, consistent with our observation. This broad range could be due to the diverse diagnostic criteria for END and the interval between evaluations (Del Bene et al., 2012; Duan, Fu, et al., 2015; Duan, Sun, et al., 2015; Jiang et al., 2019; Kamouchi et al., 2011; Ois et al., 2008; Ryu et al., 2012; Tamura et al., 2013; Yamada et al., 2004; Yamamoto et al., 2010; Zhou et al., 2018).

Several studies have revealed predictors of END in SSI, including age, sex (female), diabetes, blood pressure variability, severe and large infarcts, initial NIHSS score, presence of penetrating artery disease, and inflammatory markers (Del Bene et al., 2012; Duan, Sun, et al., 2015; Jin et al., 2023; Kim et al., 2015; Serena et al., 2001; Terasawa et al., 2008; Yamamoto et al., 2010; Zhang et al., 2022; Zhou et al., 2018). Our study on SSI types is consistent with previous studies (Duan, Fu, et al., 2015; Duan, Sun, et al., 2015). Compared with other stroke types, the key mediating factor for END in patients with SSI is an increase in the infarct volume (Cho et al., 2009; Yamada et al., 2004). The perforator artery is an end artery lacking collateral supply; therefore, the branching mode and condition of the involved perforator artery determine the increase in infarct size and the final infarct size (Hallevi et al., 2012; Kim et al., 2008; Serena et al., 2001; Yamada et al., 2004). Consistent with the results of previous studies, higher blood glucose levels were associated with END in patients with SSI who received IVT. Hyperglycemia is common in patients with acute stroke, including those with diabetes or previously undiagnosed diabetes, affecting up to 50% of patients. There is strong evidence that high blood glucose levels are associated with the damaging effects of the acute phase of stroke and are an independent predictor of larger infarct size, poorer clinical outcomes, and higher mortality (Cardoso et al., 2018; Fuentes et al., 2018; Long et al., 2021; Yang et al., 2017). We found that red blood cells were associated with END. Many studies have found that red blood cell parameters, such as cell count and hematocrit, are associated with ASCVD (acute ischemic cerebral disease) risk in the general population (Hatamian et al., 2014; Paquette et al., 2021).

Most studies suggest that elevated triglyceride levels are associated with an increased risk of ischemic stroke, but there are conflicting associations between HDL‐C levels and stroke (Haibin et al., 2022; Yuan et al., 2023). Our study found that a low HDL‐C level was a risk factor for END. HDL has important anti‐atherosclerotic functions, including antioxidant effects. Some studies found that impaired HDL antioxidant activity is associated with more severe acute ischemic stroke and may also predict poorer functional outcomes in these patients (Tziomalos et al., 2019).

Consistent with previous studies, the risk factors for END differed between pSSI and dSSI (Duan, Fu, et al., 2015; Duan, Sun, et al., 2015; Wen et al., 2013; Yamamoto et al., 2010). Our study found a lower incidence of END in dSSI than in pSSI. Additionally, there was a positive correlation between HDL‐C and blood glucose levels in patients with pSSI. No risk factor was associated with END in patients with dSSI. This difference may be because dSSI and pSSI have different pathologies (Tamura et al., 2013; Wen et al., 2013; Yamada et al., 2004). It is believed that dSSI is a small vessel disease caused by lipid hyaluronic disease (Wardlaw et al., 2013). END is a chronic and stable pathological condition with a rare occurrence; therefore, no predictive factors have been identified (Yamada et al., 2004). pSSI is mostly caused by maternal atherosclerosis blocking the perforator artery (Caplan, 1989; Nam et al., 2021; Wardlaw et al., 2013), and END may occur by progressing from near occlusion to complete occlusion through additional thrombosis or plaque bleeding or by an embolus spreading from the unstable plaque to the distal branch (Hallevi et al., 2012; Nam et al., 2021; Rashid et al., 2022; Terasawa et al., 2008; Wardlaw, 2005; Yan et al., 2023).

We studied the risk factors for END in patients with branch atheromatous disease (BAD) after thrombolysis. Our findings were consistent with those of previous studies regarding the risk factors for the occurrence of END in patients with BAD without thrombolysis. It has been suggested that thrombolysis is not efficacious in preventing END in patients with BAD. Vynckier et al. (2021) found that dual antiplatelet therapy was associated with a reduction in the risk of END (*p* = .04); however, the IVT treatment did not show this effect. Other studies have shown that IVT does not prevent patients with BAD from developing END (Marcelinus et al., 2022; Nagakane et al., 2008; Park et al., 2016). Early research by Yamamoto et al. (1999) found that coagulation and fibrinolysis activation and platelet function were significantly increased in patients with END, suggesting that combined antiplatelet and anticoagulation therapy may be used to prevent the occurrence of END in patients with acute cerebral infarction.

This study had certain limitations. This was a retrospective cross‐sectional study; because of the limitations of the cross‐sectional design, causality could not be proven with our data alone. Moreover, this study had a small sample size and used either conventional MRA or CTA. Without a high‐resolution MRA, determining the location and distribution of the actual perforating artery is not possible. Finally, a relatively sensitive definition of END was used, which could have resulted in an overestimation of the prevalence of END.

## 5 CONCLUSIONS

The proportion of END in patients with SSI in the MCA territory after IVT was high; therefore, the SSI type, HDL‐C, blood glucose, and red blood cell levels should be monitored closely. Additionally, the frequency and predictors of SSI in the territory of the MCA were different between pSSI and dSSI.

## AUTHOR CONTRIBUTIONS

Meng Zhao and Xuemin Zhong were responsible for the concept and design of the study, data collection, and the first and final drafts of the paper. Jian Wang, Lanying He, and Jiaxiu Du were responsible for the study concept and design, data analysis, and interpretation. All the authors have read and approved the final manuscript for publication.

## CONFLICT OF INTEREST STATEMENT

The authors declare no conflicts of interest.

### PEER REVIEW

The peer review history for this article is available at https://publons.com/publon/10.1002/brb3.3283.

## Data Availability

The data sets used and/or analyzed in the current study are available from the corresponding author upon reasonable request.
